# Correlation between motor symptoms, cognitive function, and optical coherence tomography findings in Parkinson’s disease

**DOI:** 10.5935/0004-2749.2024-0049

**Published:** 2024-10-31

**Authors:** Leonardo Provetti Cunha, Pedro Nascimento Martins, Luiza Cunha Martins, Fernanda Mara do Nascimento Almada, Nádia Shigaeff, Daniel Oliveira de Araújo, Luiz Guilherme Marchesi Mello, Mário Luiz Ribeiro Monteiro, Peter J. Snyder, Thiago Cardoso Vale

**Affiliations:** 1 Faculdade de Medicina, Universidade Federal de Juiz de For a, Juiz de Fora, MG, Brazil; 2 Hospital de Olhos de Juiz de Fora, Juiz de Fora, MG, Brazil; 3 Division of Ophthalmology, Faculdade de Medicina, Universidade de São Paulo, São Paulo, SP, Brazil; 4 Department of Biomedical and Pharmaceutical Sciences, University of Rhode Island, Kingston, Rhode Island, USA; 5 Department of Neurology and of Surgery (Ophthalmology), Alpert Medical School of Brow University, Providence, Rhode Island, USA

**Keywords:** Parkinson’s disease, Tomography, optical coherence, Neurodegenerative diseases, Cognitive dysfunction, Cognition, Motor perception, Visual acuity, Retina

## Abstract

**Purpose:**

This study aimed to evaluate the total macular thickness as well as the thickness of the inner and outer retinal layers in patients with Parkinson’s disease. It also aimed to verify the correlation of these parameters with motor symptoms and cognitive function.

**Methods:**

A total of 46 eyes of 23 patients with Parkinson’s disease and 40 eyes of 20 healthy controls were included in the study. The patients’ cognitive, functional, and nonmotor symptoms were evaluated using the Katz Index of Independence and Pfeffer’s Activities of Daily Living, Mini-Mental State Examination, Frontal Assessment Battery, Schwab and England Staging Scales, and Movement Disorders Society Nonmotor Symptoms Scale. The macular thickness measurements obtained via total, inner, and outer optical coherence tomography were recorded. Furthermore, the correlation of the parameters of optical coherence tomography with cognitive, functional, and nonmotor symptoms was assessed.

**Results:**

The scores of the Katz Index of Independence and Pfeffer’s Activities of Daily Living as well as the Movement Disorders Society Nonmotor Symptoms Scale were significantly lower in patients with Parkinson’s disease than in healthy controls. Moreover, the former had greater total macular thickness. The temporal and inferior outer sectors were significantly greater for the ganglion cell complex thickness in patients. A significant correlation was observed between the total macular thickness and the Movement Disorder Society-Unified Parkinson’s Disease Rating Scale, Parte III (MDS-UPDRS-III) values. Contrarily, there was a negative correlation between the outer macular thickness and the MDS-UPDRS-III values. Meanwhile, the total macular thickness and ganglion cell complex thickness were significantly correlated with the scores of the Mini-Mental State Examination, Schwab and England Staging Scale, Frontal Assessment Battery, and Katz Index of Independence and Pfeffer’s Activities of Daily Living. In addition, the Schwab and England scale was correlated with the outer macular thickness.

**Conclusion:**

The total and inner macular thicknesses at the temporal and inferior outer sectors were greater in patients with Parkinson’s disease than in the control group. These findings indicate that macular thickness may be greater in those with Parkinson’s disease, particularly when associated with mild motor symptoms. In addition, the parameters of the total, inner, and outer optical coherence tomography were significantly associated with motor and nonmotor symptoms as well as cognitive function impairment.

## INTRODUCTION

Parkinson’s disease (PD) is one of the most common neurodegenerative disorders and is characterized by the loss of dopaminergic neurons in the substantia nigra pars compacta and the accumulation of aggregates of the protein alpha-synuclein in several nuclei of the central nervous system^(1)^. PD patients typically present with motor and nonmotor symptoms. Cognitive impairment is one of the most prevalent and incapacitating nonmotor symptoms in PD^(2)^.

The retina has emerged as a potential biomarker in many neurodegenerative diseases^(3^,^4)^. Some studies have suggested that retinal involvement can occur in the very early stages of PD, even before the onset of the first symptoms^(5)^. Patients with PD experience a wide range of visual disturbances throughout the disease course, such as reading difficulties, double vision, and difficulty performing tasks that require vision assistance^(6)^. According to some studies, up to a quarter of PD patients suffer from hallucinations, particularly those of a visual nature, as the disease progresses^(6)^. Previous studies have also correlated the severity of PD motor symptoms with poorer visual acuity in these patients^(7)^. Other visual symptoms may also be present, such as decreased contrast perception, difficulty in color discrimination, changes in movement perception, and hypometric saccade^(8)^. Most of these visual deficits occur through mechanisms associated with changes involving retinal cells and layers^(6)^. Autopsy studies conducted on patients with PD have reported that these patients have reduced dopamine concentration in the retina compared with those without PD^(9)^. Furthermore, studies using electroretinogram have demonstrated that patients with PD exhibit reduced electrical activity in the retina compared with controls^(10)^.

In the last decade, optical coherence tomography (OCT) has proved to be a valuable method for the detection of axonal loss in the retinal tissue of patients with neurodegenerative diseases, being most of the studies done in Alzheimer’s disease (AD). Its ability to generate accurate and reproducible measurements of the retina provide new insights into the use of this technology as a potential biomarker for neurodegenerative diseases.

Despite the evidence that PD affects the retina, the literature results are conflicting. While some studies demonstrated that the OCT retinal thickness measurements are affected in PD^(11^-^14)^, others failed to confirm this involvement^(15^-^19)^. Conversely, cognitive impairment and dementia are more prevalent in more severe PD cases^(2)^. As already demonstrated in patients with PD, it is expected that the greater the degree of cognitive impairment, the more pronounced the thickness reduction of the retinal layers, indicating the greater severity of the disease^(20)^.

To the best of our knowledge, only one recently published study has evaluated the structural measurements of the retina and their correlations with motor symptoms and cognitive performance in PD^(21)^. However, cognitive changes are exclusively assessed using the Mini-Mental State Examination (MMSE), which evalua-tes various cognitive domains, such as temporal and spatial orientation, ganglion cell layer (GCL), inner plexiform layer (IPL), and outer nuclear layer (ONL) were thinner in patients with PD, significantly decreased as the Hoehn-Yahr (H-Y) stage progressed. The study also found that the GCL and IPL thicknesses were correlated with motor symptoms, which were evaluated using the Movement Disorder Society-Unified Parkinson’s Disease Rating Scale III (MDS-UPDRS III) and nonmotor symptom assessment^(21)^. Therefore, in addition to using the aforementioned instrument for cognitive performance assessment, this study also proposes to expand this assessment by evaluating executive function, which encompasses performance in inhibitory control, mental flexibility, planning and organization, verbal fluency, working memory, and sustained and selective attention. Furthermore, this study aimed to assess total macular thickness as well as the inner and outer retinal layer thicknesses and to verify the correlation between these measurements with motor symptoms and cognitive performance.

## METHODS

A total of 46 eyes of 23 PD patients and 40 eyes of 20 healthy controls (HCs) were included in this study. The patients were recruited from the Movement Disorders Division of the Neurology Service at the University Hospital of the Federal University of Juiz de Fora, Minas Gerais, Brazil.

PD was diagnosed based on the United Kingdom Parkinson Disease Society Brain Bank Criteria^(17)^. The patients’ clinical data, including age, schooling years, time of the diagnosis, duration of symptoms and treatments, and current and previous medications, were obtained at evaluation or from the medical records.

The exclusion criteria were age <18 years; poor collaboration; evidence of brain damage; previous history of acute myocardial infarction, stroke, and renal failure; poorly controlled systemic arterial hypertension (i.e., systolic and diastolic blood pressure exceeding 120 and 80 mmHg, respectively); diabetes mellitus (i.e., fasting blood glucose <130 mg/dL and glycated hemoglobin (A1C) <7%); and illiteracy. Patients who previously underwent ocular surgery (except for uncomplicated cataract six months earlier) and those with macular disease, glaucoma, or other optic neuropathies, intraocular pressure >21 mmHg, spherical refraction > ± six diopters and cylinder refraction > ± three diopters, media opacities, were also excluded.

Disease severity was measured using the motor status of the MDS-UPDRS-III in the ON and OFF states as well as the H-Y and Schwab and England (S&E) staging scales.

Neurological assessment and part III-UPDRS evaluation were conducted in the same appointment, whereas cognitive, functional, and nonmotor symptom assessments were conducted together with the ophthalmological examinations. The interval between the appointments was up to 2 weeks.

### Optical coherence tomography

The PD patients and HCs underwent a pertinent ophthalmic evaluation and OCT scanning with the use of Swept-Source OCT (SS-OCT, DRI OCT Triton, Topcon Corp., Tokyo, Japan).

For each eye, a set of three high-quality images was obtained in a raster pattern covering a 6 × 6-mm area for the optic nerve head acquisition protocol, with a scan density of 512 × 256 pixels (100,000 A scans/s). The total, inner, and outer macular thickness measurements were based on three high-definition SS-OCT images centered at the fovea in a raster pattern covering a 7 × 7-mm area with a scan density of 512 (vertical) × 256 (horizontal) pixels. The criteria for acceptable 3D SS-OCT fundus images were no large eye movements, defined as an abrupt shift completely disconnecting a large retinal vessel, consistent signal intensity level across the scan, and no black bands caused by blinking throughout the acquisition time, as previously described^(3)^.

**Figure 1:**
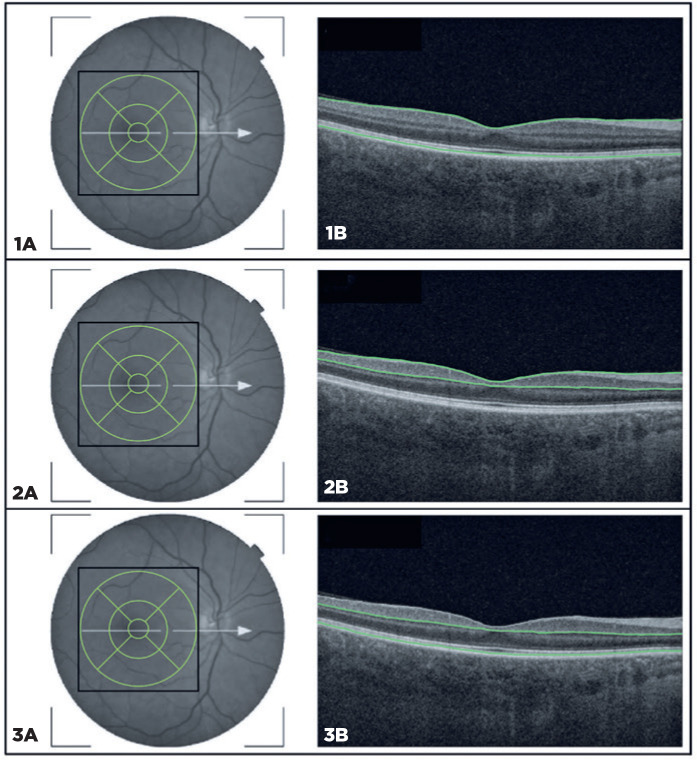
Representative optical coherence tomography (OCT)-scanned area with nine sectors ETDRS grid (6 × 6mm) centered on the fovea (1A, 2A, and 3A) and OCT B-scans (1B, 2B, and 3B). The green lines represent the boundaries of the retinal layers identified in the segmentation process. Figure 1B: the total macular thickness, with the green lines representing the internal limiting membrane and the posterior boundary of the photoreceptor outer segment layer. Figure 2B: the ganglion cell layer complex (GCC), which comprises the macular retinal nerve fiber layer and the posterior limit of the ganglion cell layer/inner plexiform layer. Figure 3B: the outer macular thickness, representing the anterior boundary of the inner nuclear layer and the posterior boundary of the photoreceptor outer segment layer.

The OCT software automatically calculated the total, inner, and outer macular thickness parameters based on the Early Treatment Diabetic Retinopathy Study (ETDRS) map (Figure 1). This map is divided into nine sectors, including the fovea (1 mm), the inner sectors (3 mm around the center of the fovea), and the outer sectors (6 mm around the center of the fovea); each of them, inner and outer, divided into superior, nasal, temporal, and inferior sectors. The software automatically calculated the thicknessof these nine sectors in microns (µm) and the average macular thickness. Furthermore, it automatically delimited the boundaries between the anatomical inner and outer retinal layers in the macular area. An experienced examiner evaluated the boundaries that were automatically defined by the software in each scan and repeated the entire acquisition when boundary errors were present to avoid manual segmentation corrections. The inner macular thickness parameter comprises the macular retinal nerve fiber layer (mRNFL) and GCL/IPL thickness and is referred to as the retinal ganglion cell complex (GCC). Outer macular thickness measurements were calculated as the difference between the total and inner macular thicknesses and included the following layers: inner nuclear layer (INL), outer plexiform layer (OPL), ONL, and photoreceptor layers (i.e., inner and outer segments) (Figure 1).

### Cognitive assessment

All the patients’ cognitive, functional, and nonmotor symptoms were assessed by a neuropsychologist. For this purpose, a comprehensive assessment protocol covering general cognitive aspects, nonmotor symptoms, and functionality was developed.

To assess functionality, the Katz Index of Independence and Pfeffer’s Activities of Daily Living Index were used. The Katz Index evaluates the level of dependence or independence of the participants in basic activities of daily living, such as dressing, bathing, and feeding. The scores range from 0 to 6, with higher scores indicating greater independence.

The Pfeffer Index also assesses the level of dependence or independence but in instrumental activities of daily living, such as shopping, meal preparation, financial management, and staying informed about community events. The scores range from 0 to 3, with higher scores indicating greater dependence.

The Movement Disorder Society Nonmotor Symptoms Scale (MDS-NMS) assesses a wide range of symptoms unrelated to physical movement but significantly impacting the quality of life, including neuropsychiatric, gastrointestinal, urinary, sensory, cognitive, and autonomic symptoms. The total score is obtained by summing the scores of all the items assessed, reflecting the severity and frequency of the symptoms.

In the cognitive assessment, the MMSE and the Frontal Assessment Battery (FAB) are employed.

The MMSE is widely recognized in the scientific literature for the assessment of various cognitive areas, such as temporal and spatial orientation, long-term memory, language, attention, and calculation. The scores range from 0 to 30, with higher scores indicating better cognitive function. The FAB is employed to assess executive functions, such as planning, organization, decision--making, mental flexibility, and inhibitory control. Scores are assigned to each subitem, reflecting performance in the evaluated executive functions.

### Statistical analysis

When discrete, the clinical and sociodemographic variables from the PD patients and HCs were expressed as absolute and relative frequencies. For the analysis of cognitive results, a statistical analysis was conducted using the total scores of each individual and for each instrument. Numerical variables were expressed as medians and quartiles or means and standard deviations as appropriate. Descriptions for OCT measurements were performed similarly, using the eye as the unit of analysis.

The Wilcoxon rank-sum test was employed to compare the performance in cognitive and functional assessments between the PD patients and HCs. In addition, OCT data from the patients were compared using generalized estimating equation (GEE) models. The GEE analysis was conducted to compensate for the inclusion of both eyes of the same participant. Therefore, the GEE models were used to adjust for within-patient and inter-eye correlations.

The link function for the Gaussian distribution with OCT parameters was used as the dependent variable and either the group (PD patients or HC) or the cognitive, functional, or disease manifestation scales as predictors. The measurement scales with a perceptible ceiling effect, namely, MMSE and FAB, had lost points as predictors, indicating worse cognitive performance. Spearman’s rank correlation was employed to assess the correlation between the OCT thickness measurements and the cognitive, functional, and nonmotor symptom assessments scores. *P*<0.05 was considered to indicate statistical significance. All analyses were conducted using the R software version 4.2.0.

## RESULTS

A total of 86 eyes, including 46 eyes of 23 PD patients and 40 eyes of 20 HCs, were included in the study. Table 1 summarizes the patients’ clinical and demographic data. No significant differences were observed in age and schooling years between the groups. Participants with pseudophakia were not included in either the PD or the control group. The mean time since the diagnosis of PD was 8 years. The mean MDS-UPDRS III score was 35. Most patients (n=13) were classified as H-Y stage 2, and the mean S&E score was 80.

**Table 1:** Clinical and demographic data of Parkinson’s disease (PD) patients and healthy controls (HCs)

Characteristic	PD n=23	Healthy controls n=20	p-value
Age	59 (8)	58 (10)	0.76^*^
Mean (SD)	45-76	43-81	
Minimum-Maximum			
Male sex	15 (65%)	9 (45%)	0.045^**^
Scholarity (years)			
1-3	0 (0%)	2 (10%)	0.079^&^
4-7	7 (37%)	4 (20%)	0.164^&^
>7	12 (63%)	14 (70%)	0.280^&^
Time since diagnosis			
Median [25%-75%]	8 [6-11]		
Minimum-Maximum	2-23		
UPDRS III			
Mean (SD)	35 (13)		
Minimum-Maximum	12-61		
Hoehn-Yahr			
1	3 (14%)		
2	13 (62%)		
3	3 (14%)		
4	1 (4.8%)		
5	1 (4.8%)		
Schwab & England			
Median [25%-75%]	80 [65-90]		
Minimum-Maximum	40-100		

SD= standard deviation; UPDRS= Unified Parkinson Disease Rating Scale. P-values <0.05 were considered statistically significant.

* Student’s t-test,

** Chi-square test.

& Fisher’s exact test.

The results of the cognitive, nonmotor, and functional assessments are presented in table 2. The PD patients had significantly lower Katz Index, Pfeffer’s Index, and MMSE scores than the HCs. No statistical difference was observed between the groups in terms of the FAB scores. The MDS-NMS was not obtained for the HC group.

**Table 2:** Cognitive and functional assessments of Parkinson’s disease (PD) patients and healthy controls (HCs)

Variables	PD n=23	HC n=20	p-value^1^
Katz			
Median [25%-75%]	5 [4-6]	6 [6-6]	<0.001
Minimum-Maximum	0-6	6-6	
Pfeffer			
Median [25%-75%]	1 [0-6]	0 [0-0]	0.008
Minimum-Maximum	0-20	0-4	
Mini-Mental State Examination			
Median [25%-75%]	25 [22-26]	28 [26-28]	0.018
Minimum-Maximum	16-29	17-29	
Frontal Assessment Battery			
Median [25%-75%]	11 [10-14]	16 [9-17]	0.087
Minimum-Maximum	7-18	5-18	
MDS-NMS			
Median [25%-75%]	11 [7-17]	-	NA
Minimum-Maximum	0-20	-	

MDS-NMS= Movement Disorders Society Nonmotor Symptoms Scale.

1 Wilcoxon rank-sum test. NA= not applicable. P-values <0.05 were considered statistically significant and presented in bold.

### Macular parameters in patients with PD and HCs

**Figure 2:**
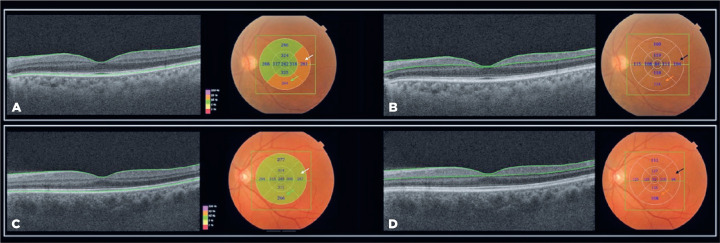
Representative scans of a patient with Parkinson’s disease (PD) (A and B) and a control (C and D). A: OCT B-scan and total macular thickness ETDRS-map of a PD patient. The green lines represent the boundaries of the neurosensory retina. B: The same PD patient as depicted in A, with OCT B-scan showing the ganglion cell layer complex (GCC) thickness ETDRS-map measurements. The green lines represent the boundaries of the internal limiting membrane (ILM) and the ganglion cell layer/inner plexiform layer (GCL/IPL). C: OCT B-scan and total macular thickness ETDRS-map of the control. D: The same control subject as shown in C, with OCT B-scan displaying the GCC thickness ETDRS-map measurements. Notably, the measurements of the outer temporal and outer inferior sectors of both total macular thickness (white and green arrows indicating the temporal outer and the inferior outer, respectively) and GCC thickness (black and orange arrows indicating the temporal outer and inferior outer, respectively) were thicker in PD patients than in the control.

The total macular thickness was greater in PD patients than in HCs, with the differences being significant in the temporal and inferior outer sectors (p=0.031 vs. p=0.015, respectively) (Table 3 and Figure 2). For the GCC thickness values, the temporal and inferior outer sectors were also significantly greater in PD patients than in HCs (p=0.018 vs. p=0.049) (Table 4 and Figure 2). For the outer macular measurements, the thicknesses in all OCT sectors were greater in PD patients than in HCs. However, the differences were not significant (Table 4).

**Table 3:** Total macular thickness measurements (mm) of Parkinson’s disease (PD) patients and healthy controls (HCs) via optical coherence tomography, divided into nine sectors according to the ETDRS-map

Parameter (mm)	PD n=46	HC n=40	p-value^1^
Average thickness			
Mean [25%-75%]	278 [267-285]	272 [266-280]	0.183
Fovea			
Mean [25%-75%]	231 [225-251]	245 [239-254]	0.265
Superior Inner			
Mean [25%-75%]	309 [298-319]	306 [302-315]	0.977
Temporal Inner			
Mean [25%-75%]	298 [284-306]	294 [288-302]	0.544
Inferior Inner			
Mean [25%-75%]	307 [294-318]	304 [299-311]	0.463
Nasal Inner			
Mean [25%-75%]	308 [295-315]	310 [302-316]	0.652
Superior Outer			
Mean [25%-75%]	270 [263-283]	267 [259-276]	0.185
Temporal Outer			
Mean [25%-75%]	260 [250-266]	250 [245-257]	0.031
Inferior Outer			
Mean [25%-75%]	263 [255-274]	254 [247-264]	0.015
Nasal Outer			
Mean [25%-75%]	282 [274-293]	279 [271-291]	0.504

Statistically significant values (p<0.05) are in bold.

1 Generalized estimating equations.

**Table 4:** Inner (GCC) and outer macular thickness measurements (mm) of Parkinson’s disease (PD) patients and healthy controls (HCs) via optical coherence tomography, divided into nine sectors according to the ETDRS-map

Parameters (µm)	PD n=46	HC n=40	p-value^1^
GCC thickness			
Average thickness			
Mean [25%-75%]	105 [100-110]	103 [98-108]	0.381
Superior inner			
Mean [25%-75%]	116 [112-125]	120 [114-124]	0.231
Temporal inner			
Mean [25%-75%]	107 [102-111]	109 [102-113]	0.946
Inferior inner			
Mean [25%-75%]	117 [111-123]	119 [113-124]	0.903
Nasal inner			
Mean [25%-75%]	111 [106-119]	116 [111-121]	0.077
Superior outer			
Mean [25%-75%]	105 [98-111]	104 [96-109]	0.205
Temporal outer			
Mean [25%-75%]	93 [86-97]	89 [83-94]	0.018
Inferior outer			
Mean [25%-75%]	105 [99-109]	102 [94-105]	0.049
Nasal outer			
Mean [25%-75%]	119 [110-124]	117 [110-122]	0.878
Outer retinal thickness			
Average			
Mean [25%-75%]	173 [169-175]	169 [166-174]	0.645
Superior inner			
Mean [25%-75%]	194 [190-198]	189 [183-197]	0.375
Temporal inner			
Mean [25%-75%]	192 [186-198]	188 [184-197]	0.594
Inferior inner			
Mean [25%-75%]	190 [185-194]	186 [183-192]	0.324
Nasal inner			
Mean [25%-75%]	198 [191-203]	193 [190-199]	0.513
Superior outer			
Mean [25%-75%]	168 [164-174]	165 [162-171]	0.375
Temporal outer			
Mean [25%-75%]	166 [161-170]	162 [160-169]	0.800
Inferior outer			
Mean [25%-75%]	158 [153-164]	157 [153-160]	0.448
Nasal outer			
Mean [25%-75%]	169 [162-172]	167 [161-169]	0.781

Statistically significant values (p<0.05) are in bold.

1 Generalized estimating equations.

### Relationship between retinal imaging parameter changes and motor and nonmotor symptoms

No correlation was observed between the total macular and GCC thickness measurements and the time since the PD diagnosis (Table 5). However, a negative correlation was found between the time since the PD diagnosis and the outer macular thickness measurements (average thickness, superior outer, superior inner, temporal inner, and nasal inner) (Table 5).

**Table 5:** Correlations between the optical coherence tomography parameters and motor and nonmotor symptoms

Variable	Time Since Diagnosis		UPDRS III	p-value	MDS Nonmotor		Schwab & England	p-value
	Estimate (95% CI)	p-value	Estimate (95% CI)		Estimate (95% CI)	p-value	Estimate (95% CI)	
Total macular thickness (in µm)								
Average thickness	-0.43 (-1.59-0.73)	0.470	-0.42 (-0.82--0.02)	0.038	0.12 (-0.68-0.92)	0.772	0.34 (0.006-0.67)	0.046
Fovea	-0.34 (-2.77-2.10)	0.787	-0.42 (-0.90-0.06)	0.084	0.73 (-0.76-2.22)	0.337	0.37 (-0.13-0.87)	0.142
Superior outer	-0.81 (-1.70-0.09)	0.077	-0.35 (-0.73-0.04)	0.076	0.17 (-0.63-0.96)	0.684	0.39 (0.15-0.63)	0.0015
Superior inner	-0.35 (-1.78-1.07)	0.627	-0.49 (-1.05-0.06)	0.079	-0.05 (-1.02-0.92)	0.919	0.49 (0.08-0.89)	0.019
Inferior inner	-0.18 (-1.93-1.58)	0.842	-0.49 (-1.03-0.04)	0.071	0.06 (-0.94-1.06)	0.906	0.39 (-0.08-0.85)	0.101
Inferior outer	0.26 (-1.26-1.78)	0.739	-0.44 (-0.86--0.02)	0.040	0.19 (-0.75-1.13)	0.689	0.19 (-0.21-0.58)	0.358
Temporal outer	-0.78 (-1.64-0.08)	0.075	-0.30 (-0.73-0.13)	0.176	0.16 (-0.79-1.12)	0.739	0.30 (-0.008-0.61)	0.056
Temporal inner	-0.80 (-2.15-0.56)	0.250	-0.52 (-0.99--0.04)	0.033	0.04 (-1.03-1.11)	0.938	0.51 (0.08-0.93)	0.020
Nasal inner	-0.53 (-2.13-1.07)	0.516	-0.54 (-1.11-0.02)	0.060	-0.06 (-1.04-0.93)	0.909	0.50 (0.02-0.98)	0.042
Nasal outer	-0.38 (-1.72-0.96)	0.580	-0.50 (-0.99--0.01)	0.046	0.007 (-0.82-0.83)	0.987	0.33 (-0.08-0.74)	0.118
GCC thickness (in µm)								
Average thickness	0.29 (-0.45-1.03)	0.437	-0.23 (-0.52-0.06)	0.116	0.008 (-0.36-0.38)	0.968	0.15 (-0.09-0.39)	0.226
Fovea	0.52 (-0.56-1.60)	0.344	0.06 (-0.21-0.32)	0.673	0.37 (-0.24-0.98)	0.236	0.05 (-0.16-0.27)	0.623
Superior outer	-0.06 (-0.66-0.54)	0.848	-0.17 (-0.41-0.08)	0.179	0.03 (-0.35-0.42)	0.875	0.19 (0.05-0.33)	0.010
Superior inner	0.68 (-0.35-1.71)	0.195	-0.26 (-0.77-0.25)	0.313	-3e-05 (-0.46-0.46)	>0.999	0.22 (-0.16-0.60)	0.262
Inferior inner	0.52 (-0.58-1.62)	0.354	-0.29 (-0.77-0.19)	0.235	-0.04 (-0.52-0.44)	0.883	0.20 (-0.19-0.58)	0.318
Inferior outer	0.66 (-0.40-1.72)	0.223	-0.30 (-0.60-0.008)	0.056	0.08 (-0.53-0.69)	0.790	0.08 (-0.20-0.36)	0.569
Temporal outer	-0.10 (-0.60-0.40)	0.687	-0.17 (-0.37-0.03)	0.099	3e-04 (-0.44-0.44)	0.999	0.11 (-0.06-0.27)	0.197
Temporal inner	0.31 (-0.45-1.08)	0.423	-0.24 (-0.60-0.11)	0.183	-0.05 (-0.37-0.27)	0.768	0.18 (-0.11-0.47)	0.229
Nasal inner	0.65 (-0.51-1.80)	0.271	-0.25 (-0.79-0.29)	0.369	-0.09 (-0.58-0.40)	0.729	0.22 (-0.20-0.63)	0.302
Nasal outer	0.33 (-0.63-1.29)	0.499	-0.30 (-0.69-0.09)	0.126	-0.08 (-0.56-0.41)	0.757	0.16 (-0.15-0.47)	0.304
Outer retinal thickness (in µm)								
Average thickness	-0.57 (-1.11--0.02)	0.040	-0.23 (-0.47-0.01)	0.065	0.10 (-0.50-0.70)	0.748	0.19 (0.005-0.37)	0.044
Fovea	-0.88 (-2.28-0.52)	0.220	-0.48 (-0.88--0.08)	0.020	0.36 (-0.74-1.46)	0.522	0.32 (-0.02-0.66)	0.066
Superior outer	-0.58 (-1.03--0.14)	0.010	-0.22 (-0.45-0.01)	0.067	0.12 (-0.48-0.73)	0.688	0.20 (0.04-0.36)	0.016
Superior inner	-0.88 (-1.45--0.32)	0.002	-0.28 (-0.61-0.06)	0.102	-0.06 (-0.87-0.74)	0.875	0.26 (0.04-0.48)	0.018
Inferior outer	-0.28 (-0.80-0.24)	0.287	-0.19 (-0.39-0.02)	0.075	0.10 (-0.43-0.62)	0.717	0.10 (-0.08-0.28)	0.280
Inferior inner	-0.58 (-1.41-0.26)	0.175	-0.24 (-0.54-0.06)	0.120	0.09 (-0.65-0.82)	0.819	0.18 (-0.04-0.40)	0.101
Temporal outer	-0.52 (-1.05-0.01)	0.054	-0.16 (-0.44-0.12)	0.259	0.15 (-0.47-0.77)	0.630	0.19 (-0.001-0.39)	0.051
Temporal inner	-1.01 (-1.80--0.22)	0.012	-0.30 (-0.69-0.10)	0.137	0.08 (-0.82-0.98)	0.858	0.32 (0.02-0.62)	0.035
Nasal outer	-0.48 (-1.08-0.11)	0.111	-0.25 (-0.49--0.005)	0.045	0.07 (-0.57-0.70)	0.840	0.16 (-0.05-0.37)	0.137
Nasal inner	-1.02 (-1.71--0.33)	0.004	-0.34 (-0.70-0.008)	0.055	0.02 (-0.81-0.84)	0.971	0.28 (0.02-0.53)	0.035

MDS-NMS= Movement Disorders Society Nonmotor scale; UPDRS III= motor status of the Movement Disorders Society-Unified Parkinson Disease Rating Scale. Estimates and 95% confidence intervals (CI) were obtained using generalized estimating equations.

Furthermore, there was a significant direct and positive correlation between total macular thickness (average thickness, inferior outer, temporal inner, and nasal outer) and MDS-UPDRS-III scores. Contrarily, there was a negative correlation between the outer macular thickness parameters and MDS-UPDRS-III scores (fovea and nasal outer). Also, no correlation was observed between the MDS-UPDRS-III scores and GCC thickness measurements (Table 5).

NMSS was not found to be correlated with the total, inner, and outer macular thickness measurements. The S&E scale scores were found to have a significant positive correlation with the total macular thickness parameters (average thickness, superior outer, superior inner, temporal inner, and nasal inner), the superior outer sector in the GCC thickness measurements, and the outer macular thickness measurements (average thickness, superior outer, superior inner, temporal inner, and nasal inner) (Table 5).

### Relationship between retinal imaging parameters changes and cognitive assessment

The Katz’s Index scores were found to have a significant positive correlation with the total macular thickness (superior outer and nasal outer) and the GCC thickness (superior outer and temporal outer) (Table 6). Similarly, Pfeffer’s Index scores were correlated with the total macular thickness (superior outer, temporal inner, and nasal inner) and the GCC thickness (superior outer and temporal outer) (Table 6). The FAB scores were correlated with the total macular thickness (nasal outer sector) and the GCC thickness (average thickness, superior outer, temporal outer, nasal outer, inferior inner, and temporal inner). Finally, significant correlations were observed between the lower MMSE scores and reductions in the total macular thickness (superior outer, temporal outer) and GCC thickness (temporal outer sector) (Table 6).

**Table 6:** Correlations between the optical coherence tomography parameters and cognitive assessment

Variable	Katz		Pfeffer		FAB (Each lost point)		MMSE (Each lost point)	
	Estimate (95% CI)	p-value	Estimate (95% CI)	p-value	Estimate (95% CI)	p-value	Estimate (95% CI)	p-value
Total macular thickness (in µm)								
Average thickness	1.90 (-0.94-4.74)	0.190	-0.72 (-1.56-0.12)	0.091	-1.69 (-3.83-0.45)	0.122	-0.87 (-2.26-0.51)	0.217
Fovea	0.37 (-5.91-6.65)	0.907	0.09 (-1.79-1.96)	0.925	-0.14 (-2.71-2.43)	0.917	1.91 (-1.22-5.04)	0.231
Superior outer	2.71 (0.34-5.08)	0.025	-0.87 (-1.51--0.22)	0.008	-1.40 (-3.39-0.59)	0.167	-1.17 (-2.25--0.09)	0.033
Superior inner	2.00 (-1.63-5.63)	0.281	-0.95 (-2.06-0.16)	0.094	-2.01 (-5.00-0.98)	0.189	-1.18 (-2.74-0.37)	0.137
Inferior inner	0.69 (-3.79-5.16)	0.763	-0.62 (-1.97-0.73)	0.366	-2.37 (-5.10-0.37)	0.090	-0.88 (-3.60-1.84)	0.527
Inferior outer	0.52 (-3.23-4.28)	0.785	-0.27 (-1.53-0.99)	0.677	-1.29 (-3.28-0.70)	0.204	0.04 (-2.23-2.30)	0.976
Temporal outer	1.79 (-1.02-4.60)	0.211	-0.64 (-1.44-0.15)	0.113	-1.45 (-3.69-0.80)	0.207	-1.38 (-2.64--0.13)	0.031
Temporal inner	2.63 (-0.79-6.04)	0.131	-1.02 (-1.95--0.10)	0.030	-2.40 (-5.14-0.33)	0.085	-1.28 (-2.91-0.34)	0.122
Nasal inner	1.92 (-1.97-5.82)	0.333	-0.98 (-2.16-0.20)	0.103	-2.36 (-5.54-0.82)	0.146	-1.02 (-2.69-0.65)	0.231
Nasal outer	3.04 (0.18-5.89)	0.037	-1.06 (-1.93--0.20)	0.016	-2.13 (-4.12--0.14)	0.036	-1.12 (-2.34-0.10)	0.073
GCC thickness (in µm)								
Average thickness	1.13 (-0.64-2.89)	0.212	-0.39 (-1.01-0.23)	0.218	-1.07 (-1.97--0.17)	0.020	-0.36 (-1.24-0.51)	0.414
Fovea	-1.07 (-3.83-1.69)	0.447	0.33 (-0.52-1.19)	0.445	-0.02 (-1.29-1.26)	0.977	1.22 (-0.45-2.89)	0.153
Superior outer	1.80 (0.74-2.87)	<0.001	-0.51 (-0.83--0.18)	0.002	-1.03 (-1.83--0.23)	0.012	-0.55 (-1.25-0.14)	0.120
Superior inner	0.29 (-2.84-3.42)	0.857	-0.37 (-1.56-0.81)	0.538	-1.22 (-2.92-0.48)	0.160	-0.58 (-2.10-0.93)	0.450
Inferior inner	0.71 (-2.38-3.81)	0.650	-0.44 (-1.55-0.67)	0.437	-1.62 (-3.12--0.12)	0.034	-0.44 (-2.09-1.20)	0.597
Inferior outer	0.34 (-2.41-3.08)	0.811	-0.07 (-1.02-0.88)	0.890	-0.86 (-1.75-0.03)	0.058	0.40 (-1.32-2.11)	0.651
Temporal outer	1.08 (-0.04-2.21)	0.059	-0.36 (-0.68--0.04)	0.026	-0.87 (-1.71--0.03)	0.042	-0.66 (-1.30--0.01)	0.045
Temporal inner	1.09 (-1.09-3.28)	0.328	-0.49 (-1.28-0.30)	0.224	-1.18 (-2.22--0.14)	0.026	-0.52 (-1.45-0.40)	0.268
Nasal inner	0.68 (-2.59-3.95)	0.685	-0.47 (-1.67-0.73)	0.441	-1.42 (-3.21-0.38)	0.122	-0.38 (-1.86-1.10)	0.614
Nasal outer	2.13 (0.05-4.21)	0.044	-0.67 (-1.40-0.06)	0.073	-1.31 (-2.35--0.27)	0.014	-0.73 (-1.49-0.03)	0.059
Outer retina (in µm)								
Average thickness	0.71 (-1.28-2.69)	0.486	-0.37 (-0.91-0.17)	0.177	-0.81 (-2.34-0.73)	0.303	-0.53 (-1.27-0.21)	0.159
Fovea	1.44 (-2.71-5.59)	0.496	-0.25 (-1.47-0.98)	0.695	-0.13 (-2.25-1.99)	0.903	0.69 (-1.18-2.57)	0.470
Superior outer	0.82 (-1.06-2.71)	0.393	-0.39 (-0.90-0.13)	0.140	-0.54 (-2.02-0.94)	0.475	-0.64 (-1.29-0.007)	0.053
Superior inner	1.61 (-0.60-3.82)	0.154	-0.62 (-1.25-0.02)	0.056	-1.00 (-2.94-0.93)	0.308	-0.63 (-1.44-0.18)	0.127
Inferior outer	0.10 (-1.72-1.93)	0.911	-0.23 (-0.69-0.23)	0.322	-0.61 (-2.00-0.77)	0.384	-0.38 (-1.17-0.40)	0.336
Inferior inner	-0.11 (-2.86-2.64)	0.938	-0.21 (-0.94-0.52)	0.568	-0.88 (-2.70-0.93)	0.339	-0.46 (-1.91-1.00)	0.538
Temporal outer	0.63 (-1.41-2.68)	0.543	-0.31 (-0.89-0.28)	0.302	-0.73 (-2.32-0.86)	0.367	-0.75 (-1.50-0.005)	0.052
Temporal inner	1.47 (-1.45-4.39)	0.323	-0.55 (-1.42-0.31)	0.207	-1.33 (-3.41-0.75)	0.209	-0.78 (-1.83-0.28)	0.150
Nasal outer	0.77 (-1.14-2.69)	0.429	-0.44 (-0.94-0.06)	0.087	-1.10 (-2.45-0.26)	0.114	-0.42 (-1.26-0.42)	0.326
Nasal inner	1.14 (-1.37-3.65)	0.373	-0.55 (-1.24-0.15)	0.123	-1.15 (-3.29-0.99)	0.291	-0.67 (-1.68-0.35)	0.197

FAB= Frontal Assessment Battery; MDS-NMS= Movement Disorders Society Nonmotor Symptoms Scale; MMSE= Mini-Mental State Examination; Estimates and 95% confidence intervals (CI) were obtained using generalized estimating equations.

## DISCUSSION

In the present study, the Katz and Pfeffer’s Index scores as well as the MMSE scores were significantly lower in PD patients than in HCs. This finding indicates that cognitive impairment is detectable in the early stages of the disease. The risk factors for dementia in PD include an H-Y score >2, MMSE score <29, and advanced age^(22^,^23)^. In fact, most patients (78.3%) in the present study were classified into stage 2 or higher on the H-Y and had a mean score of 25 on the MMSE. Furthermore, as previously described in the literature regarding retinal structural and functional abnormalities^(24^,^25)^, a significant cognition impairment was found in PD patients.

Nonmotor symptoms may precede the classical motor manifestations that are used for the clinical diagnosis of PD^(26)^. Moreover, cellular abnormalities in the central nervous systems and peripheral tissues possibly begin decades before the manifestation of the motor symptoms^(27)^. These abnormalities include loss of dopaminergic neurons, α-synuclein misfolding, phosphorylation and aggregation, as well as mitochondrial dysfunction^(26)^. Similarly, as occurs in the brain, lower neurotransmitter levels, alpha-synuclein deposition, disruption of iron homeostasis, chronic inflammation, and oxidative stress may be associated with retinal abnormalities in PD^(28)^. Thus, the mainly affected retinal cells are believed to be the amacrine, bipolar, interplexiform, and retinal ganglion cells.

Despite the evidence of retinal involvement in PD, the capability of OCT to reliably detect retinal changes in patients is still unclear. While some studies have demonstrated a decrease in total and inner retinal thicknesses (i.e., mRNFL, GCL/IPL)^(11^,^19)^, others have not been able to confirm these findings^(19)^. Interestingly, both the total and inner retinas were found to have greater thicknesses in PD patients than in HCs, with statistical significance in the temporal and inferior outer sectors. Previous studies have reported an impairment of the ganglion cells in neurodegenerative diseases, such as AD and PD itself. However, the alterations of the IPL in these conditions have been poorly discussed. Owing to its reflectivity, the IPL is usually measured in conjunction with the GCL, and these two layers, along with the macular RNFL, form the GCC. The IPL is located between the GCL and the INL, containing the synapses between bipolar cell axons, ganglion cell dendrites, and amacrine cell processes.

Previous studies have reported a reduction in the thickness of the GCC in PD^(29^,^30)^. The GCC layer combines the mRNFL and the GCL/IPL. Thus, the GCL is formed by ganglion cells, whose axons give rise to mRNFL, and the dendrites originate from the IPL layer. The dopaminergic amacrine cells connect in networks with the ganglion cells in the image formation transmission process and along with the RNFL projections converge to the optic nerve. In patients with PD, a decrease in dopamine concentration^(14^,^15^,^28^-^32)^ results in a reduced dopaminergic stimulus to the ganglion cells, which consequently leads to atrophy of the ganglion cells and their nerve fibers. This causes thinning of the GCL and mRNFL layers. Histological analysis of the retina of PD patients reinforces consistent signs of retinal degeneration preferentially affecting the inner retinal layers due to abnormal accumulation of α-synuclein^(21)^. Similarly, it is suggested that PD’s decreased inner retinal layers’ thicknesses are consistent with the loss of retinal dopaminergic amacrine cells, and it correlates with the severity of the disease.

Our findings indicate that the thickness measurements of the inner retinal layers were greater in PD patients, particularly in the temporal and inferior outer sectors. A possible explanation for the GCL and IPL might be increased in PD is neuroplasticity. In PD, the increased thicknesses of the GCL and IPL may reflect compensatory neuroplastic changes, such as dendritic remodeling, synaptic reorganization, or gliosis, a compensatory response in the face of dopaminergic neuronal loss^(11^,^32^-^34)^. Thus, it is possible that in the early stages of PD, dopaminergic deficiency may contribute to glutamate overproduction, cellular dysregulation, and changes in retinal architecture and measurements by OCT^(21)^. However, the exact cause may not be completely understood. Our findings must be interpreted with caution. Also, further studies are warranted to confirm these findings.

The present study found a negative correlation between the time since the diagnosis and the outer macular thickness measurements, indicating that the longer the diagnostic time, the thinner this layer will be. Similarly, Gunay and Usta^(35)^ reported that the outer retinal thickness was negatively correlated with more prolonged disease duration. The duration and stage of the disease appear to be associated with the most severe cases at more advanced disease stages. Wang et al.^(21)^ showed that the inner and outer retinal thicknesses significantly decreased as the MDS-UPDRS III score and H-Y stage increased. The study also demonstrated the total macular thickness and the outer retinal thickness in many ETDRS sectors were correlated with the MDS-UPDRS-III scores. However, it is worth mentioning that the participants selected in our study were mostly in the mild stages of the disease, mainly H-Y stage 2. Thus, these findings suggest that in the early stages of PD, the changes in retinal thickness are mild or even absent and that thickening of the outer retina can be observed, involving both the OPL and retinal pigment epithelium, as reported in other studies. Retinal thickening may be a result of an inflammatory process secondary to the deposition of α-synuclein, metabolic impairment, or compensatory mechanism preceding the degenerative process^(21^,^36)^.

Our study demonstrated a significant correlation between the OCT parameters and nonmotor symptom assessments scores. The S&E scale scores were found to be correlated with the total, inner, and outer OCT thickness measurements. Cognitive function impairment is one of the most crucial nonmotor symptoms owing to its significant impact on the quality of life and activities of daily living. It may be regarded as a promising prodromal indicator in PD. Only a limited number of studies have examined the correlation between cognitive impairment and OCT parameters in PD. Wang et al.^(21)^ showed that GCL and IPL thicknesses correlate with nonmotor symptom assessment scores. They suggest that the macular inner retinal layer tends to be much thinner in patients with PD exhibiting nonmotor symptoms. In our study, the scores of the MMSE, Katz Index, Pfeffer’s Index, and FAB were correlated with the total and GCC thickness measurements.

In summary, the total and inner macular thicknesses at the temporal and inferior outer sectors were greater in PD patients than in HCs. This finding suggests that the macular thickness is greater in patients with PD, particularly when associated with mild motor symptoms. In addition, we demonstrated a significant correlation between the total, inner, and outer OCT parameters and the motor and nonmotor symptoms as well as cognitive function impairment.
